# Macroporous epoxy-based monoliths for rapid quantification of *Pseudomonas aeruginosa* by adsorption elution method optimized for qPCR

**DOI:** 10.1007/s00216-020-02956-3

**Published:** 2020-10-03

**Authors:** Lisa Göpfert, Julia Klüpfel, Charlotte Heinritz, Martin Elsner, Michael Seidel

**Affiliations:** Institute of Hydrochemistry, Chair of Analytical Chemistry and Water Chemistry, Technical University of Munich, Marchioninistr. 17, 81377 Munich, Germany

**Keywords:** Monolith filtration, *Pseudomonas aeruginosa*, Culture-independent, Adsorption elution

## Abstract

*Pseudomonas aeruginosa* contaminations in tap water systems have caused severe health problems in both hospital and household settings. To ensure fast and reliable detection, culture-independent methods are recommendable. However, the typically low cell number in water samples requires sample enrichment prior to analysis. Therefore, we developed and optimized an adsorption elution method using monolithic adsorption filtration and subsequent centrifugal ultrafiltration that can be combined with culture-independent detection methods. The principle of adsorption of *Pseudomonas aeruginosa* by hydrophobic and ionic interactions was studied in modified epoxy-based monoliths. Optimized conditions (5-L initial sample volume at pH 3 filtered for 30 min through hydrolyzed monoliths (MAF-OH) and eluted with beef extract glycine buffer at pH 9.5) achieved a recovery of 67.1 ± 1.2% and a concentration factor of 10^3^. For the first time, we therefore present a culture-independent approach for rapid enrichment and subsequent molecular biological quantification of *P. aeruginosa* by qPCR from tap water samples by monolithic adsorption filtration. The total enrichment and quantification process takes 4 h. This work further stresses the versatility of the monolithic adsorption filtration and its possibilities as a concentration tool for culture-independent analytics of pathogenic bacteria in the environment.

Graphical abstract

**Electronic supplementary material:**

The online version of this article (10.1007/s00216-020-02956-3) contains supplementary material, which is available to authorized users.

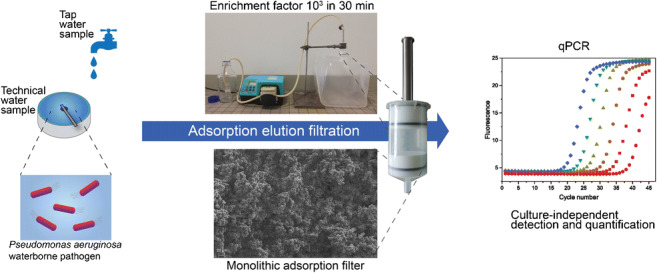

## Introduction

Providing safe drinking water — or lowering risk for workers in contact with technical water — nowadays requires very sensitive analytical techniques to detect and rapidly quantify pathogenic bacteria. Therefore, it is necessary to concentrate bacteria from large water sample volumes prior to subsequent analysis by cultivation-independent methods. Here, traditional sterile filtration has its disadvantages as the filters tend to clog quickly due to matrix components when large water sample volumes are processed. Monolith-based filtration offers an attractive alternative. Monoliths are homogeneous stationary phases with high porosity. They can be comprised of organic or inorganic material, most often silica [1]. While silica monoliths have a high surface, their limited pH working range (stability in a pH range from 2 to 8) often excludes them from applications targeting bacterial concentration using adsorption-elution processes [2]. Organic monoliths, in contrast, are easy to synthesize and stable at even extreme pH, which compensates for the lower surface compared with silica monoliths [1, 3]. An overview of different enrichment methods for microorganisms is given in Table 1.

**Table 1:** Overview of different enrichment methods for microorganisms [4–7]

Method	Advantages	Disadvantages
Centrifugation	Easy handling	Loss of non-viable cells possible Matrix may also be concentrated Often only size specific
Immunofiltration	Very specific due to antibody interaction	Cost intensive due to antibodies More steps needed for synthesis
Immunomagnetic separation	Very specific due to antibody interaction	Cost intensive due to antibodies Low sample volumes
Flocculation	Can enrich multiple microorganisms at once	Time intensive Based on unspecific interactions
Membrane filtration	Automation possible Defined size range Different materials possible	Dead-end filtration may result in clogged filters
Monolithic filtration	Easily adaptable due to functionalization Easy and fast synthesis of unfunctionalized filters Used for different microorganisms Applicable over a wide range of pH values, flow rates, and matrices	Sometimes complicated functionalization

One of the applications of an organic monolith is the monolithic adsorption filtration which has already been applied efficiently in concentrating different bacteria and viruses from water [8, 9]. An epoxy-based monolith with free epoxy groups on the filter surface can be synthesized within 1 h with a defined pore size of 21 μm [10]. The production costs of the unfunctionalized monolithic adsorption filters (MAFs) are below 1 € per single use MAF. The free epoxide groups allow functionalization for desired adsorption properties in an easy and fast manner. Different functionalization types of the MAFs have been published, such as hydrolyzed MAFs (MAF-OH) [9] with hydroxyl groups on the MAF surface, MAFs with diethyl aminoethyl groups on the surface (MAF-DEAE) [11], and MAFs with the antibiotic polymyxin B immobilized on the pore surface (MAF-PmB) [10]. The retention of bacterial cells on the monolith is based on different interactions such as ionic or hydrophobic interactions, hydrogen bonds, or van der Waals forces. The individual composition of these forces depends on the analyte and the MAF functionalization. Due to the monolith’s structure (macroporous with no mesopores), MAF filtration of microorganisms can be performed at high flow rates and with large sample volumes. An overview of different MAF types is given in Table 2. In this study, only BigMAF were used and are referred to as MAF or MAF disks, but with respect to the desired sample volumes also other MAF types could be applied. Additionally, batch or continuous sampling and automation of the whole process is possible and dead-end as well as crossflow applications have been developed [8]. The possibility to process high volumes enables high concentration factors that are crucial for a rapid detection of low cell numbers in environmental samples. While this adsorption elution process is commonly used for the enrichment of viruses, it was first applied to the enrichment of *Salmonella* sp. and *Escherichia coli* as early as 1976 using DEAE cellulose columns [12] and adapted for MAFs for *Escherichia coli* [10] and *Legionella* sp. [9]. In this work, for the first time, an adaption towards *Pseudomonas aeruginosa* concentration has been done.

**Table 2:** Overview of previously published MAF types and their properties. *d*, diameter in mm; *h*, height in mm [8, 10, 11, 13, 14]

MAF type	Flow rate	Max. sample volume and filtration time	Elution volume	Cartridge type	Modification
microMAF *d* = 4.5 *h* = 6.0	10 mL min^−1^	300 mL in 30 min	200 μL	Glass	-OH -Polymyxin B
miniMAF *d* = 3.7 *h* = 11.8	0.7–34 mL min^−1^	1000 mL in 33 min	1 mL	Glass	-Antibody
BigMAF *d* = 38.6 *h* = 10.0	1 L min^−1^	100 L in 100 min	20 mL	Plastic	-OH -DEAE
miniMAF *d* = 10.0 *h* = 10.0	50 mL min^−1^	1 L in 20 min	1 mL	Plastic	-OH -DEAE

*P. aeruginosa* is a Gram-negative, biofilm-forming bacterium causing different diseases such as pneumonia, wound infections, and sepsis. It is present in most environmental water bodies, intrinsically resistant to a number of standard antibiotics, and further known to easily acquire new resistances, which makes infections often difficult to treat [15, 16]. Because of this, *P. aeruginosa* is especially dangerous for immunosuppressed people, e.g., patients in hospitals or people with chronic illnesses. Due to the biofilm formation, contaminations in the tap water system, e.g., of water meters or at the water tap, are tenacious once they have formed. This is because of the reduced susceptibility of bacteria in a biofilm towards disinfection or antimicrobial agents [17, 18]. In consequence, *P. aeruginosa* is among the opportunistic premise plumbing pathogens which are of increasing research importance as they are prone to survive and even thrive in these systems [19, 20]. In recent years, several contaminations of *P. aeruginosa* in tap water systems have gathered public attention. In 2014, several water meters to be installed at kindergartens, hospitals, and retirement homes in Hamburg, Germany, showed contaminations and needed to be changed [21]. Additionally, several outbreaks of nosocomial infections linked to *P. aeruginosa* from water systems have been registered: In France, cases in an oncohematology pediatric unit (2005) [22], and in the ear, nose, and throat department of a hospital (2014) [23]. Additional reports stem from Northern Ireland in several neonatal units (2011–2012) [24] and in the USA in a neonatal intensive care unit (2017) [25]. For contaminated sites, a level of > 150 CFU 100 mL^−1^ has been reported [22]. However, *P. aeruginosa* is also found in technical water systems such as cooling water towers [26] and in several other settings [27]. To prevent their spreading and possible infection outbreaks, thorough monitoring is desirable. In case of a contamination, rapid detection by culture-independent methods is crucial to cut incubation time short, thus allowing for prompt disinfection measures.

Standard operating procedures for detection of *P. aeruginosa* from water samples rely on culture methods, which take a long time to give dependable results (up to 48 h) and which may even have difficulties in capturing the correct number of the target pathogens because they may “hide” in biofilms. Especially with contaminations in tap water systems, time is essential to prevent further infections. Here, culture-independent analysis methods can give reliable results within hours instead of days. Culture-independent methods are molecular biological assays such as the (quantitative) polymerase chain reaction (qPCR) or, alternatively, flow cytometry, spectroscopic methods (Raman or IR), and immunoassays. Additionally, and in contrast to culture, these methods can detect bacteria also in a viable but non-culturable (VBNC) state, which *P. aeruginosa* is known to enter if insufficient disinfection took place [28]. As these VBNC bacteria can later be resuscitated [29], they need to be captured to assess the success of disinfection measures correctly. This is of particular importance not only in drinking water systems but also in technical plants.

**Fig. 1:**
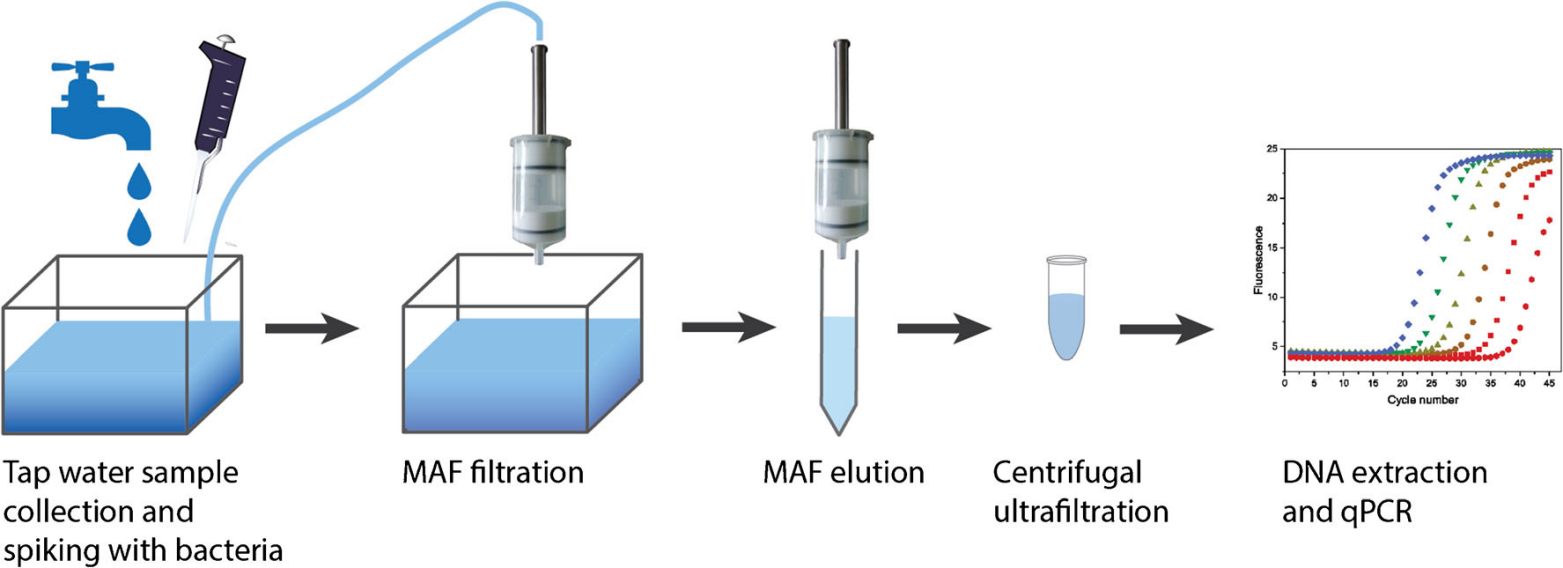
Schematic overview of the total procedure. A tap water sample is collected and spiked with bacteria. Subsequent MAF filtration and elution is followed by centrifugal ultrafiltration and DNA extraction for analysis with qPCR

To provide a sufficiently high number of bacteria for application of these culture-independent methods, we present the development and optimization of a concentration method for *P. aeruginosa* from tap water using MAF filtration in combination with centrifugal ultrafiltration. Subsequently, the feasibility of culture-independent detection was demonstrated using qPCR as an approach that avoids error-prone quantification via culture and which ensures a rapid detection. An overview of the procedure is given in Fig. 1.

## Experimental

### Materials and instrumentation

All chemicals, unless stated otherwise, were purchased from Sigma-Aldrich, subsidiary of Merck (Darmstadt, Germany). LB media, agar agar, diethylamine, glycerine, PD-tips, and sodium hypochlorite were purchased from Carl Roth (Karlsruhe, Germany). Polyglycerol-3-glycidyl ether (CL9) was obtained from ipox chemicals (Laupheim, Germany). All oligonucleotides were synthesized by Eurofins Genomics (Ebersberg, Germany), purchased in lyophilized form and dissolved in nuclease-free water. Primers for detection of the *P. aeruginosa regA* gene were designed in house. For genomic DNA extraction, the extraction kit GeneJET Genomic DNA Purification Kit by Thermo Scientific (Waltham, USA) was used. Photometric measurements were performed on a NanoPhotometer by Implen (Munich, Germany). For qPCR, the Luna® Universal qPCR Master Mix by New England Biolabs (Ipswitch, USA) was used. For all experiments, ultrapure water was used, unless stated otherwise. Nuclease-free water was purchased from Invitrogen AG (Carlsbad, USA). A bacterial isolate of *Pseudomonas aeruginosa* (ATCC27853) was received from the Bavarian Health and Food Safety Authority. Viable bacteria were handled in laboratories approved for biosafety level 2.

### Field emission scanning electron microscopy imaging

Field emission scanning electron microscopy (FESEM) images from MAF filters were obtained using a Sigma VP 300 FE-SEM (Carl Zeiss AG, Germany), run in low vacuum mode. The inner MAF structure was analyzed by cutting out 5 × 5 cm rectangles in the center of the MAF and then halving them horizontally using a scalpel. The upper side of the lower half was then measured in different magnifications and analyzed using ImageJ.

### Bacteria cultivation

*P. aeruginosa* from cryo-culture (stored at − 80 °C) was cultivated overnight (37 °C) in LB media to be used for further experiments. Appropriate dilutions were further cultivated on LB agar plates (own production) overnight (37 °C). For filtration experiments, the bacterial cell number in liquid culture of *P. aeruginosa* was calculated from optical density measurements at 600 nm (OD_600_). A calibration curve with OD measurements from fresh overnight culture was obtained by plating dilutions on agar plates and calculating the CFU mL^−1^. The empirical formula: cell number in CFU mL^−1^ = (OD_600_ − 0.0033) / (1.1244 · 10^8^) was obtained and used for all cell number calculations. All cell numbers were obtained using this process (details can be found in the Electronic Supplementary Material (ESM)).

### Monolithic adsorption filter disk production

**Fig. 2:**
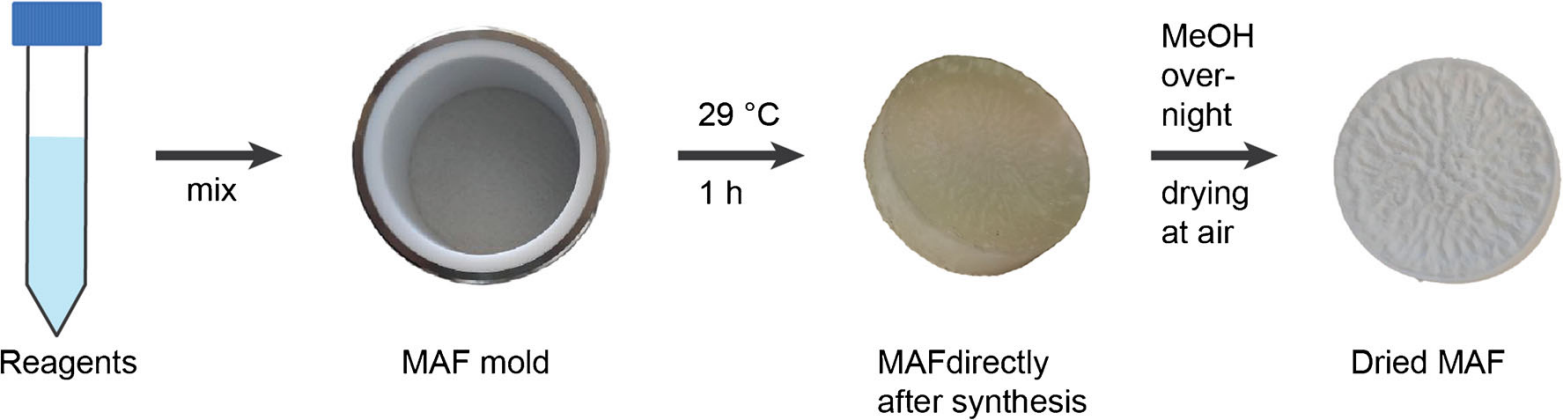
Schematic overview of the MAF production. The reagents are mixed and poured in a MAF mold. After incubation for 1 h at 29 °C, the MAF is removed from the mold and put in MeOH to end the polymerization. After removal of the MeOH, the MAFs are air-dried

**Fig. 3:**
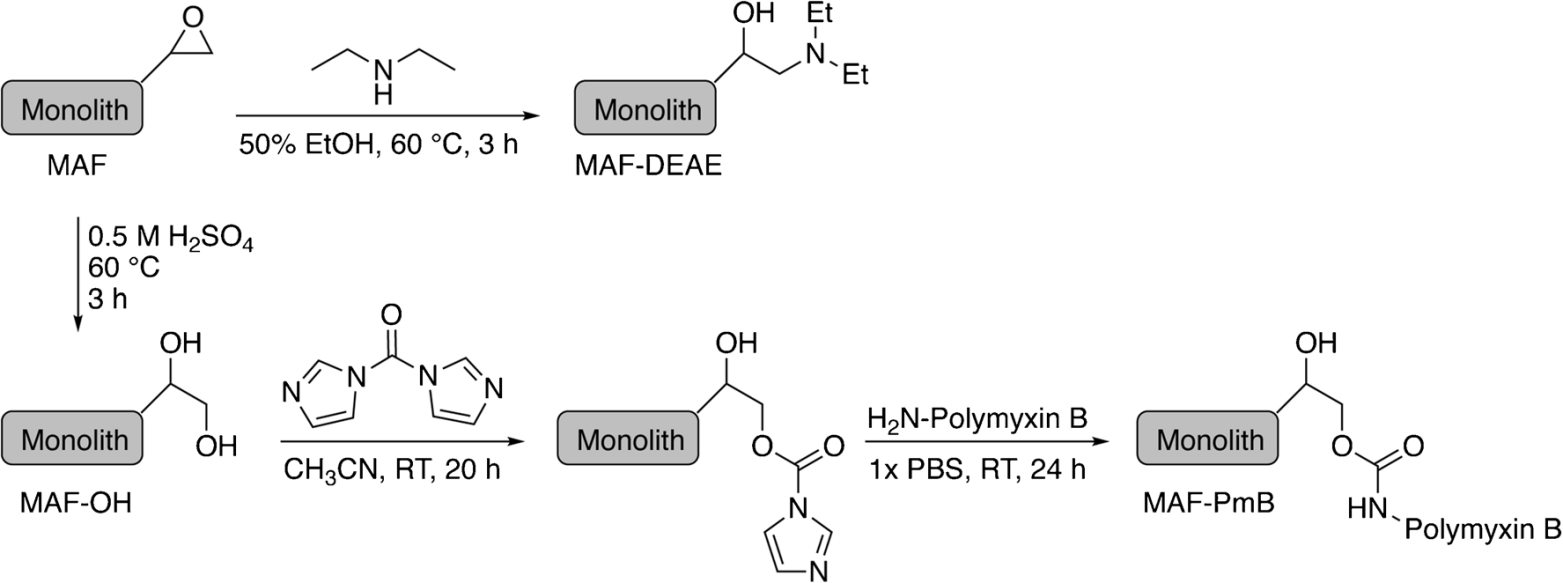
Reaction schemes for functionalization of MAFs to obtain MAF-OH, MAF-DEAE, and MAF-PmB

**Fig. 4:**
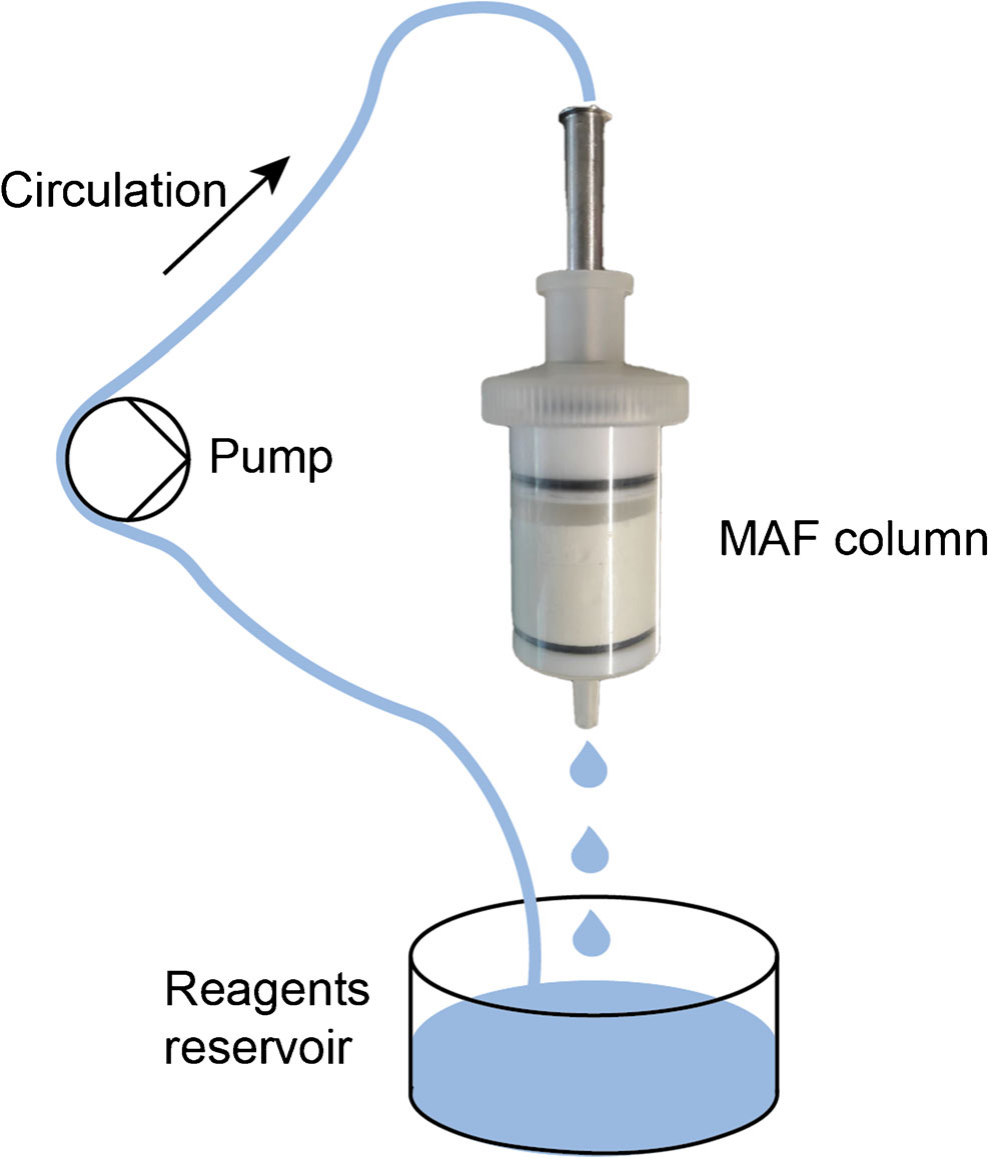
Schematic overview of the MAF functionalization. Three MAFs are functionalized at the same time by circulating the reagent solutions continuously through the MAF for the respective amount of time

MAFs were produced by self-polymerization of an epoxy-based resin [10] with minor adjustments. Briefly, a 60:40 (v/v) mixture of toluene and *tert*-butyl methyl ether was tempered at 29 °C. The catalyst trifluoride diethyl etherate (BF_3_·Et_2_O) in 1,4-dioxane (1:10 (v/v) dilution) was added to the mixture (1.25% (v/v) of the total volume) and mixed thoroughly. The monomer polyglycerol-3-glycidyl ether (CL9) (ratio 20:80 (v/v) monomer/porogen) was added and mixed thoroughly before pouring the mixture into PTFE molds (inner diameter 38.6 mm, inner height 10.0 mm) and incubating at 29 °C for 45 min. Afterwards, the MAF disks (38.6-mm diameter, 10.0-mm height) [8] were removed from the polymerization molds, stored in methanol overnight to stop the reaction, and air-dried at room temperature (Fig. 2). Reaction schemes for the different functionalizations are shown in Fig. 3. For functionalization, the MAF disks were constructed to MAF modules in 50-mL plastic dispenser tips (PD-tip) and consisting of a PTFE support plate with bore holes (diameter 2 mm), an O-ring (38.6 mm in diameter, nitrile butadiene rubber (NBR) 70), a MAF disk, and a PTFE fitting for connection to tubes. The MAF disks were washed with ultrapure water before functionalization by connecting the MAF modules to a peristaltic pump and continuously pumping ultrapure water through the system (10 min). Afterwards, the respective functionalization solutions were circulated through the system according to the functionalization procedures displayed in Table 3 (schematic overview in Fig. 4). The functionalized MAF disks were stored in ultrapure water at 4 °C until further use.

**Table 3:** Overview of functionalization procedures for MAF disks. *MAF-OH*, hydrolyzed MAFs; *MAF-DEAE*, MAFs with diethyl aminoethyl functionalization; *MAF-PmB*, MAFs with polymyxin B functionalization [9–11]

Functionalization	Functionalization solution	Circulation time	Temperature
MAF-OH	0.5 M sulfuric acid	3 h	60 °C
	Ultrapure water	15 min	RT
MAF-DEAE	10% diethylamine in EtOH/H_2_O (50/50; v/v)	3 h	60 °C
	Ultrapure water	15 min	RT
MAF-PmB	0.5 M sulfuric acid	3 h	60 °C
	Acetonitrile (ACN)	Until filtrate was clear	RT
	2 mg mL^−1^ 1,1′-carbonyl-diimidazole in ACN	Overnight	RT
	ACN, followed by PBS buffer	Until filtrate was clear	RT
	0.02 mg mL^−1^ polymyxin B in PBS buffer	24 h	RT
	PBS buffer and carbonate buffer	Until filtrate was clear	RT

### Filtration of tap water samples

Tap water samples were taken in different volumes (1-L glass bottle, 5-L polypropylene high breast bottle, or 10-L polyethylene canister) and, if necessary, pH value was adjusted to the desired pH using 37% hydrogen chloride. The desired amount of *P. aeruginosa* was spiked shortly before filtration. For all optimization experiments, cells for 10^8^ CFU L^−1^ final concentration, and for calibration experiments, the amounts for final concentrations (1·10^4^–1·10^8^ CFU/L) were added. If heat-inactivated bacteria were used, the inactivation was done at 80 °C for 30 min. A MAF module was connected to a peristaltic pump (Pumpdrive 5206 Peristaltic Pump, Heidolph, Germany) with a silicone tube (Maprene Tube, Watson-Marlow, Wilmington) and a 10-L PE canister was placed under the outlet of the MAF module to collect the filtrate. Tap water was used to flush the system and adjust the flow rate to approx. 180 mL min^−1^. Filtration was done by inserting the water inlet into the tap water sample and using the adjusted flow rate. After filtration of the whole sample, the MAF module was flushed with air and filled with elution buffer (20 mL). Elution took place in a three-step process. Each step consisted of an incubation (2 min) and elution of one-third of the elution buffer at a flow rate of approx. 110 mL min^−1^. Used elution buffers were carbonate buffer (sodium carbonate (0.150 mM) and sodium bicarbonate (0.350 mM) in ultrapure water; pH 9.6), high-salt buffer (sodium chloride (14.99 mM) and HEPES (4-(2-hydroxyethyl)-1-piperazineethanesulfonic acid) (0.500 mM) in ultrapure water; pH 7, adjusted with 32% sodium hydroxide), glycine buffer (5.00 mM in ultrapure water; pH 9.5, adjusted with 32% sodium hydroxide), beef extract glycine buffer (BEG, beef extract (30.0 g) and glycine (0.505 M) in 1 L of ultrapure water; pH 9.5, adjusted with 32% sodium hydroxide), and Pluronic® F68 solution (Pluronic® F68 (1%)).

### Centrifugal ultrafiltration and DNA extraction

Centrifugal ultrafiltration (CeUF) was performed using Vivaspin 20 Membrane 50,000 columns (Sartorius, Stonehouse, UK). The eluate of the MAF filtration was centrifugated (5000 rpm) until the final volume was 0.5–1 mL. The filter was washed with sterile PBS buffer and the volume was adjusted to 1.5 mL with PBS buffer. One milliliter was used for DNA extraction while the remaining 0.5 mL was stored at − 20 °C. DNA extraction was performed using the GeneJET Genomic DNA Purification Kit by Thermo Scientific (Waltham, USA) according to manufacturer’s guideline. Initial cell harvesting was accomplished by centrifugation of the sample (5000×*g*, 10 min). DNA extracts were stored at − 20 °C.

### qPCR

Primer sequences for *regA* detection in *P. aeruginosa* are shown in Table 4 and additional information about specificity of the primer pair including cross-reactivity experiments, qPCR procedure, and calibration experiments can be found in the ESM. The amplicon length is 141 bp. All qPCR experiments were carried out on a LightCycler 480 (Roche Diagnostics, Rotkreuz, Switzerland). The assay was performed according to the manufacturer’s guidelines. Briefly, for every reaction, 10 μL Luna Master Mix (New England Biolabs, Ipswitch, USA), 0.5 μL forward (FWD) primer (10 μM), 0.5 μL reverse (REV) primer (10 μM), and 7 μL H_2_O were combined in a master mix. To this master mix, 2 μL of the DNA sample of interest was added. Non-target controls were also included in every qPCR measurement (18 μL of the master mix described above and 2 μL H_2_O).

**Table 4:** Primers for specific amplification of *P. aeruginosa* DNA in qPCR

Target/gene	Primer	Sequence (5′→3′)	Reference
regA gene	PaRegFP	CGCAAGAGCATCGAGTACCT	This work
	PaRegRP	TAGTGCCTGCCGTGACGG	

## Results and discussion

### Rapid quality control for MAFs using FESEM images

**Fig. 5:**
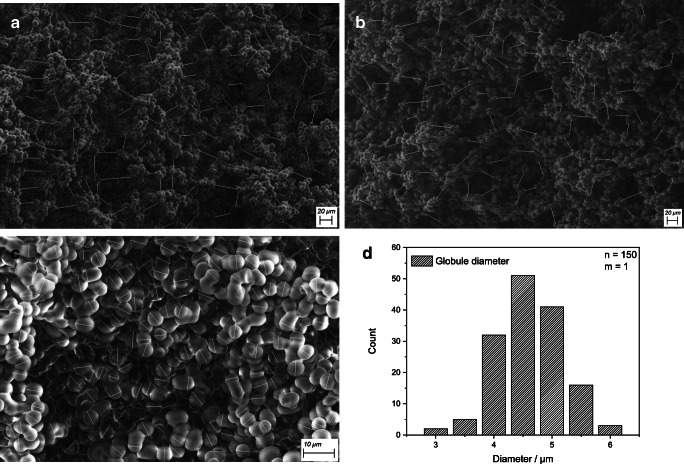
FESEM images for pristine MAF pore size (**a**), OH-functionalized MAF pore size (**b**), polymer globule diameter (**c**) with indicated measurement lines (*n* = 100 for pores and *n* = 150 for globules) and globule diameter size distribution (**d**)

To ensure successful, reproducible synthesis of MAFs, the main filter parameters should be checked for every synthesis batch. However, an easy and fast quality control for MAF has so far been missing. Mercury intrusion porosimetry was used successfully in the past, but no batch-to-batch control in a short timeframe is possible with this method. For this reason, the application of FESEM imaging analyzed with ImageJ to measure pore sizes and polymer globule diameter was tested. The following FESEM images for pore sizes (Fig. 5a) and polymer globule diameters (Fig. 5c) exemplarily show pristine MAFs from batches synthesized for this work. The observed mean pore size was 22.30 ± 6.30 μm corresponding well to the literature value of 22.5 ± 9.0 μm [10]. Functionalized MAF-OH showed a mean pore size of 22.34 ± 5.58 μm corresponding well with the mean pore size of pristine MAFs. Thus, functionalization does not change the pore size. For the mean polymer globule diameter, a value of 4.61 ± 0.56 μm was found with a relatively homogeneous size distribution (Fig. 5d), indicating a continuous and homogeneous polymerization process. Thus, FESEM measurements allow for a rapid quality assessment for different MAF batches that is important to ensure a high reproducibility of the overall method.

### Comparison of MAF functionalization types for *P. aeruginosa* filtration

**Fig. 6:**
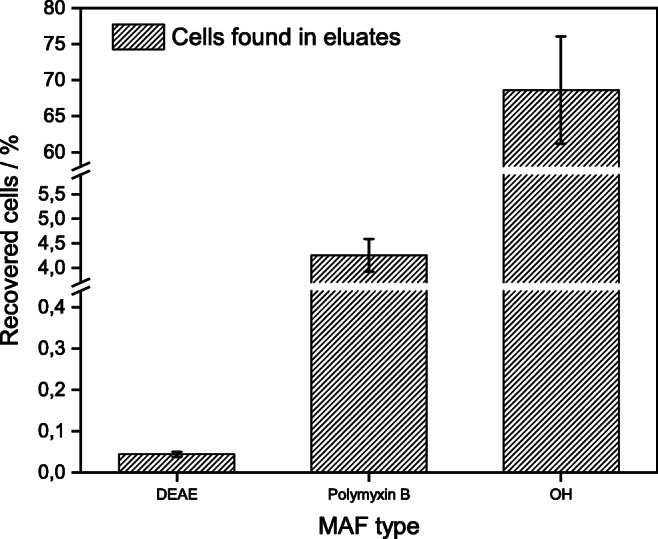
Recoveries of different MAF functionalization types: DEAE (MAF-DEAE), polymyxin B (MAF-PmB), OH (MAF-OH); *n* = 3

Three different MAF functionalization types (MAF-OH, MAF-DEAE, and MAF-PmB) were tested under the respective standard conditions to identify the MAF functionalization with the most promising results for further experiments with *P. aeruginosa*. The standard conditions were MAF-OH: 10-L sample volume, pH 3, BEG elution buffer [9]; MAF-DEAE: 1-L sample volume, pH 7, BEG elution buffer [11]; MAF-PmB: 1-L sample volume, pH 4, carbonate elution buffer [10]. An overview of recoveries is shown in Fig. 6. The number of *P. aeruginosa* cells on the MAF could not be calculated directly as no reliable concentrations of the filtrate could be determined by qPCR due to the low number of cells in the filtrate. Therefore, recovery was used to quantify the retention and elution process and, unless stated otherwise, calculated as the ratio of the total number of cells found in the eluate by qPCR after filtration and the total number of cells in the initial sample. Detailed information on how recoveries were obtained can be found in the ESM. Initial spiked concentration of *P. aeruginosa* was 10^8^ CFU L^−1^. While MAF-DEAE and MAF-PmB only show a recovery of 0.04 ± 0.01% and 4.3 ± 0.3%, respectively, MAF-OH shows a remarkably higher recovery of 68.6 ± 7.4%. Unspecific retention of bacterial cells occurs only to a very minor extent as is evident by the recovery for MAF-DEAE (0.04 ± 0.01%) which is seen as a reference for unspecific binding in this work. Additional recovery experiments with MAF-DEAE and different elution buffers as well as different filtration techniques (one-time filtration, repeated filtration of the same sample, and circulating filtration) have been carried out (data in ESM) but did not yield significantly better results. MAF-PmB uses a surface-active antibiotic as the affinity ligand while MAF-DEAE and MAF-OH make use of electrostatic interactions between the MAF surface and the bacteria for retention of the analyte. The MAF-OH seems to offer the positively charged bacteria (due to acidification) high interaction possibilities for electrostatic interaction compared with the MAF-DEAE, where almost no interaction takes place between the MAF surface and the negatively charged bacteria surface (at neutral pH due to lipopolysaccharide (LPS) structures presented on the surface). The LPS structure in *P. aeruginosa* is typical for Gram-negative bacteria consisting of lipid A, inner and outer cores, and O-antigen with variations depending on the strain (detailed information in [30]) and thus is positively charged at pH 3 and negatively charged at neutral pH. The main interaction properties are stated to be hydrogen bonds. Based on these results, MAF-OH was chosen for further optimization of the filtration process.

### Comparison of elution buffers

**Fig. 7:**
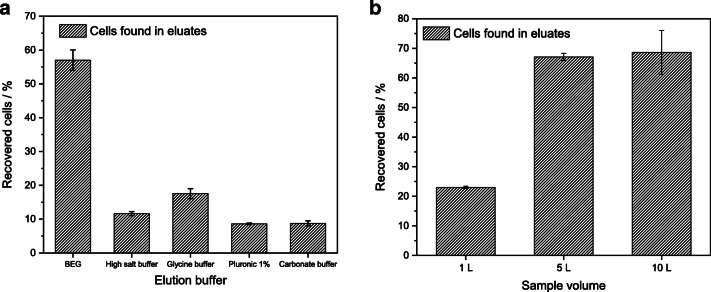
Recoveries found using different elution buffers (**a**) and initial sample volumes (**b**) for filtration with MAF-OH at pH 3, *n* = 3

Five different elution buffers (BEG buffer, high-salt buffer, carbonate buffer, glycine, and Pluronic® F68 solution) were evaluated for filtration with MAF-OH. The applied MAF process was the same for all (10-L initial sample volume, sample pH 3, 20-mL elution volume). Spiked *P. aeruginosa* cell concentration was 10^8^ CFU mL^−1^. Figure 7a shows an overview of the recoveries. While four buffers show relatively similar values with Pluronic® F68 solution (8.6 ± 0.3%), carbonate buffer (8.8 ± 0.7%), high-salt buffer (11.6 ± 0.6%), and glycine buffer (17.5 ± 1.5%), the combination of glycine with beef extract (BEG buffer) gives a significantly higher recovery of 57.0 ± 3.0%. This buffer combines the desorption properties of a change in pH (from pH 3 in the sample to pH 9.5 in the elution buffer) with proteins present therefore breaking the interactions between the cells and the monolith surface. While the change in pH changes the net charge of the bacteria, the protein uses van der Waals forces and hydrophobic interactions to remove the cells from the filter and therefore reduces the electrostatic interactions present in the adsorption process. Thus, BEG buffer was the elution buffer of choice for further experiments.

### Optimization of initial sample volume

While the original MAF-OH filtration protocol for MAF with 3.86-cm diameter and 1-cm height states 10-L initial sample volume [9], it was aimed on finding a lower sample volume with reasonably high recovery to facilitate an easier sample collection. Here, three different sample volumes (1 L, 5 L, and 10 L) were tested under the same conditions (MAF-OH, pH 3 as sample pH, and BEG elution buffer, 10^8^ CFU L^−1^ spiked *P. aeruginosa*). With increasing sample volume from 1 to 5 L, a notable increase in recovery from 23.0 ± 0.4% to 67.1 ± 1.2% was observable (Fig. 7b). One explanation for this is the distribution of the *P. aeruginosa* cells within the MAF: With a lower sample volume, the bacteria only attach to the first part of the cylindrical monolith structure while with higher sample volume, several absorption and desorption events occur before the cells finally stick to the MAF. This leads to an even distribution of the bacterial cells over the whole filter at higher sample volumes and thus higher recovery using the same elution protocol. However, a further doubling of the sample volume from 5 to 10 L only resulted in a minor increase in recovery up to 68.6 ± 7.4%. As is evident from the differing recoveries for the 10-L samples from both experiments, an interbatch variance can be seen, while the intracharge variance is relatively low. Therefore, it is important to perform quality control of each MAF charge to prevent variances in recovery values. Due to the lower filtration time, the easier handling, and the higher reproducibility (as indicated by the lower standard deviation), the optimal filtration volume for the assay was set at 5 L for *P. aeruginosa* filtration.

### Adjustment of sample pH

**Fig. 8:**
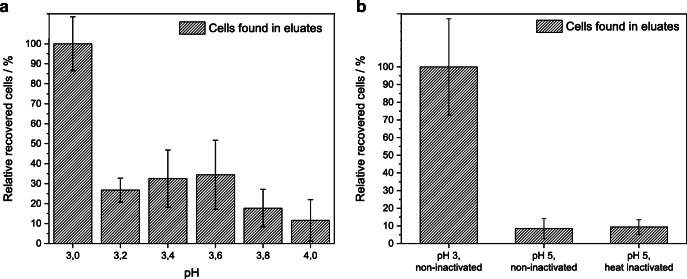
Recoveries of filtrations with different initial sample pH (**a**) and viable or heat-inactivated cells at pH 3 and pH 5 (**b**) with 5-L sample volume, MAF-OH disks, and BEG elution buffer. Recovery for pH 3 and viable cells was set at 100%, *n* = 3

To evaluate the efficiency of the filtration procedure at different pH values with MAF-OH, sample pH values between pH 3 and pH 4 (in 0.2 intervals) as well as pH 5, 6, and 7 were tested. Beforehand, culturability of *P. aeruginosa* under the tested conditions in tap water was investigated, as the goal was to find a filtration procedure that would allow subsequent detection via culture. This would then enable the use of cell culture as a confirmatory quantification method, as it is still the gold standard method despite the high time expenditure. For *P. aeruginosa*, growth on agar plates was observed for all pH values above pH 3.3 (overgrown after overnight incubation) and no growth was observed for pH values below pH 3.2 (no colonies visible), indicating a change in physiology at these pH values. This result is comparable with the result of another study investigating the effect of low pH values on the survival of *P. aeruginosa* [31] while a different study found good bacterial growth for pH higher than 3.8 [32]. For all filtrations, the cultivated *P. aeruginosa* samples were acidified before filtration and the optimized protocol (MAF-OH, 5-L initial sample volume, BEG elution buffer) was used. Spiked *P. aeruginosa* cell concentration was 10^8^ CFU L^−1^. As is visible in the relative recoveries with pH 3 set to 100% displayed in Fig. 8a, good retention and elution of the bacteria could be achieved for a sample pH of 3.0 only. Filtrations at pH 3.2 and higher showed significantly lower recoveries with the lowest recovery being at pH 4.0 (pH 3.2: 26.7% ± 5.3%; pH 3.4: 32.5% ± 12.72%; pH 3.6: 34.4% ± 15.45%; pH 3.8: 17.7% ± 8.4%; pH 4: 11.6% ± 9.3%). Relative recoveries for pH 5, pH 6, and pH 7 were 23.2%, 17.0%, and 10.6%, respectively. Thus, it was concluded that at filtration conditions where *P. aeruginosa* are culturable, no good retention and following elution is possible. The recoveries show higher values than for unspecific binding; hence, a specific interaction between the cells and the MAF surface takes place. Spiked cultivated *P. aeruginosa* which were non-culturable at pH 3 seem to be adsorbed on the surface of MAF-OH much better than culturable cells at higher pH. To evaluate the correlation between culturability and filtration efficiency, additional experiments using both heat-inactivated *P. aeruginosa* and viable cells for spiking were carried out (recoveries in Fig. 8b). For comparison, pH 3 with cells that were culturable prior to acidification was set at 100% recovery (standard deviation ± 27.2%). At a sample pH of 5, the relative recovery for heat-inactivated and culturable cells was 9.4% ± 4.1% and 8.5% ± 5.7%, respectively. This leads to the conclusion that the filtration efficiency correlates with the sample pH value but not the culturability status of the bacteria. A change in outer membrane chemistry and consequently a change in the interaction between the monolith’s surface and the outer membrane might induce the higher retainability at pH 3. As *P. aeruginosa* cells are not culturable after acidification to pH 3, a live-dead discrimination or detection via culture after filtration using the current setup is not possible, but culture-independent detection methods can be used for the successful quantification after filtration.

### Calibration of the final setup

**Fig. 9:**
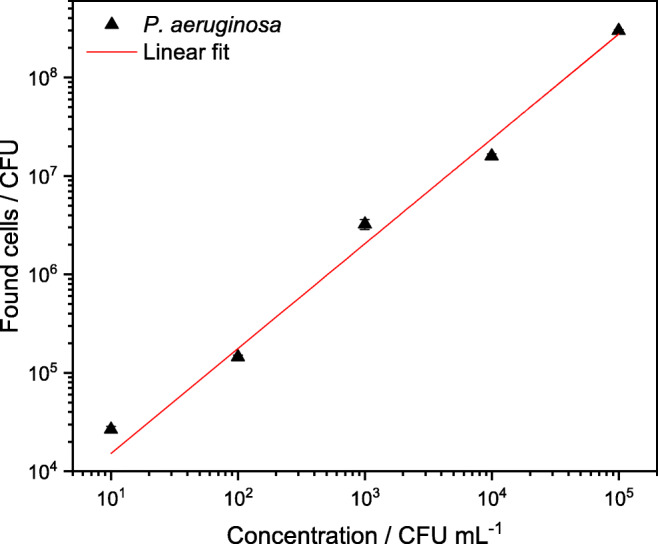
Linear range of cells found in 1.5-mL eluate against concentration in 5-L initial sample volume at pH 3 concentrated with MAF-OH and eluted with BEG buffer. *y* = (3.11 ± 0.20) + (1.07 ± 0.04) · x. *n* = 3. The error bars are smaller than the symbols indicating the values and thus not clearly visible

Based on the optimization process, a calibration using the best filtration conditions for highest recovery of *P. aeruginosa* was carried out. At a sample pH of 3, 5-L sample volume, filtration with MAF-OH, and elution with BEG buffer, five different concentrations of *P. aeruginosa* spiked in tap water (1·10^4^–1·10^8^ CFU/L) as well as a blank sample (tap water) were concentrated. The linear range of the calibration is displayed in Fig. 9. A concentration factor of 3·10^3^ in under 1 h was achieved by reducing the initial volume of 5 L to a final volume of 1.5 mL after filtration and centrifugal ultrafiltration. The filtration efficiency (recovery rate of 67.1 ± 1.2%) is included in the calibration line so that it can be used to calculate the initial sample concentration from the total number of detected cells in the eluate with the quantification method of choice.

## Conclusion

In this work, a new adsorption-elution-based method for the concentration of water samples using monolithic adsorption filtration was developed to enrich *P. aeruginosa* from tap water. Different functionalization types, sample volumes, elution buffers, and sample pH were tested to establish the most suitable conditions. The final protocol allows quantitative enrichment of *P. aeruginosa* from 5-L initial volume at pH 3 using hydrolyzed MAF and BEG elution buffer. Combined with subsequent centrifugal ultrafiltration, 5-L samples could be concentrated to a final volume of 1.5 mL, corresponding to a concentration factor of 3·10^3^. Currently, with an overall time of 4 h for the complete process used herein (under 1 h for MAF filtration and centrifugal ultrafiltration, followed by DNA extraction and qPCR quantification 3 h), this method provides a rapid detection of *P. aeruginosa* compared with culture methods which take at least 24 h. Other culture-independent detection methods such as flow cytometry [33], immunoassays [34], Raman spectroscopy [35], and Fourier transform infrared spectroscopy [36] could be used as well. Further optimization of filtration parameters will be done to allow for future use of culture as an additional confirmatory quantification method. As shown in this work, MAF filtration is a very versatile tool that can be optimized in many ways to suit one’s specific needs. It allows for the rapid enrichment of bacteria from different environmental sample types such as tap water but also process waters and other samples with challenging matrices and can be combined with various detection methods. The results presented herein are very promising for future routine application of MAF filtration in screening applications for *P. aeruginosa* in households and the environment as well as in the assessment of efficiency of disinfection measures in technical facilities and water systems.

## Electronic supplementary material

ESM 1 (PDF 446 kb)
