# Supplementary material for: Macroporous epoxy-based monoliths for rapid quantification of Pseudomonas aeruginosa by adsorption elution method optimized for qPCR
Source: Anal Bioanal Chem. 2020 Oct 3;412(29):8185–95. doi: 10.1007/s00216-020-02956-3 (PMC7584540; doi:10.1007/s00216-020-02956-3)
Supplement: Supplementary file 1 — (PDF 446 kb) [file 216_2020_2956_MOESM1_ESM.pdf]

Analytical and Bioanalytical Chemistry

Electronic Supplementary Material

Macroporous epoxy-based monoliths for rapid quantification of *Pseudomonas aeruginosa* by adsorption elution method optimized for qPCR

Lisa Göpfert, Julia Klüpfel, Charlotte Heinritz, Martin Elsner, Michael Seidel

qPCR details

qPCR was done using the Luna® Universal qPCR Master Mix by New England Biolabs. This master mix already contains all qPCR reaction components besides primers and DNA template. For setting up qPCR reactions, the master mix is thawed and per reaction, 0.5 µL of each primer at a concentration of 10 µM are added to 10 µL of master mix. Furthermore, 7 µL nuclease-free water are added so that after adding of 2 µL of template DNA, the final reaction volume of 20 µL is obtained. The pipetting of the reaction is done on ice, then the qPCR plate is sealed thoroughly and spinned briefly to remove bubbles. The used thermocycling protocol was as follows (Table S1):

Table S1 Thermocycling protocol for qPCR

Cycle step	Temperature	Time	No. of Cycles
Initial denaturation	95 °C	60 s	1
Denaturation	95 °C	15 s	45
Extension	60 °C	30 s (+ plate read)	
Melt curve	55 – 97 °C		1

qPCR was done on a Roche Light Cycler 480 using the SYBR Green I detection channel of the instrument and the LCS 480 software, version 1.5.0.39. Original qPCR plates (white, product no. 04729692001) by Roche were used. For data evaluation and Cq value determination, absolute quantification using the second derivative maximum method was done. Calibration was done on every plate using freshly diluted *P. aeruginosa* DNA calibration solutions.

DNA samples for qPCR analysis were extracted from bacteria culture (in LB medium), from tap water samples or from filtration eluates in BEG buffer. For DNA extraction and purification, the GeneJET DNA Purification Kit from ThermoFisher Scientific was used. Extraction was done from a sample volume of 1 mL. DNA extraction was done shortly after the sample generation, short-time storage was done at 4 °C. DNA purity after extraction was checked using a NanoDrop, where the A260/A280 ratio was found to be in the range of 1.8 - 2.0.

For the qPCR, an in-house developed primer set was used as presented in the experimental part. As it is derived from a primer set successfully used in recombinase polymerase amplification, functionality and specificity in qPCR was expected. This expectation was supported by BLAST analysis. The formation of an amplicon with correct length was checked by agarose gel electrophoresis after PCR.

Cross reactivity experiments with *Klebsiella pneumoniae* (with $bla_{\text{SHV-18}}$ resistance gene), *K. pneumoniae* (with $bla_{\text{CTX-M-15}}$ resistance gene) *Escherichia coli* (with $bla_{\text{CTX-M-15}}$ resistance gene), *E. coli* (with $bla_{\text{TEM-3}}$ resistance gene), *E. coli* (with $bla_{\text{CTX-M-1}}$ resistance gene), *Enterococcus faecalis*, *Bacillus subtilis*, *Penicillium italicum*, and *Aureobasidium pullulans* were negative as seen from the Cq values (Table S2). These data were confirmed by agarose gel electrophoresis where no amplicons could be detected for all the species mentioned above.

Table S2 Cq values for cross reactivity experiments

Organism or control	Cq value
Non target control	33.01
Positive control	19.44
K. pneumoniae (with $bla_{\text{SHV-18}}$ resistance gene)	32.96
K. pneumoniae (with $bla_{\text{CTX-M-15}}$ resistance gene)	32.14
E. coli (with $bla_{\text{TEM-3}}$ resistance gene)	30.18
E. coli (with $bla_{\text{CTX-M-1}}$ resistance gene)	34.62
E. coli (with $bla_{\text{CTX-M-15}}$ resistance gene)	33.06
E. faecalis	30.63
B. subtilis	32.33
P. italicum	32.32
A. pullulans	34.71

For calibration of the qPCR, a DNA sample extracted from a highly concentrated *P. aeruginosa* culture was used. The concentration of the calibration DNA standard was determined by NanoDrop measurement and serial dilutions were prepared for calibration, starting from 1 ng μL^{-1}. Figure S1 shows exemplary amplification curves obtained with calibration solutions.

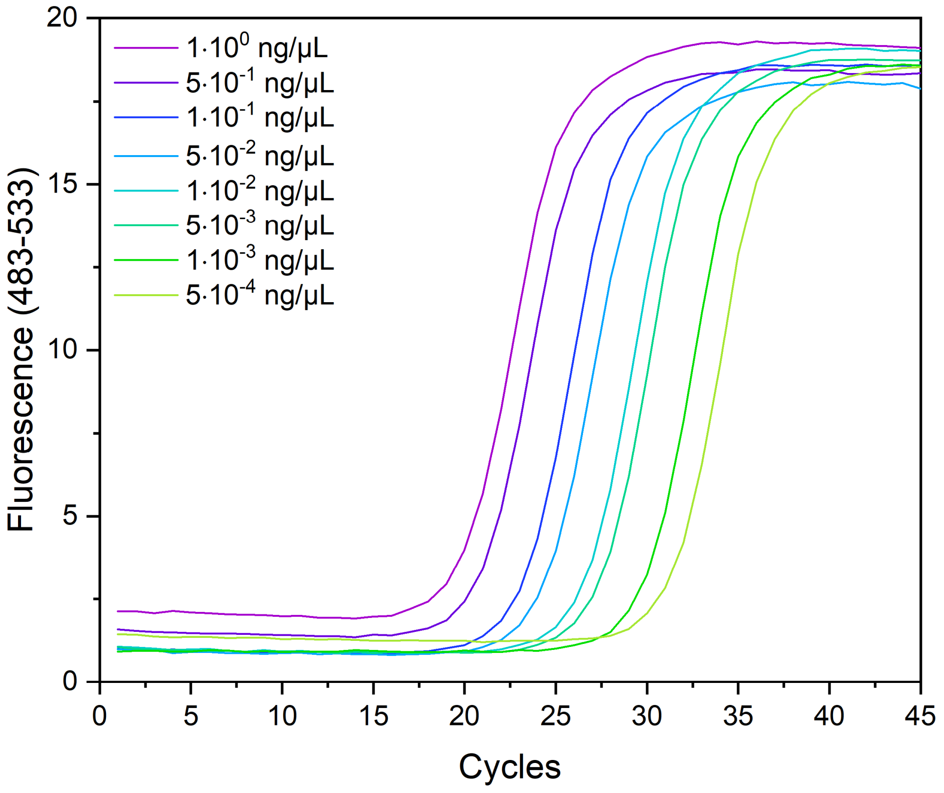

Fig. S1 Exemplary amplification curves for P. aeruginosa DNA

For some qPCR runs, an increase of fluorescence in the no target controls could be seen at high cycle numbers (approx. 35). This increase in fluorescence could be assigned to the formation of primer dimers by melting curve analysis as shown in Figure S2 and agarose gel electrophoresis. To avoid primer dimers from influencing the qPCR results, the calibration range was chosen such that the Cq value of the lowest calibration concentration was significantly lower than the one seen for some of the no target controls. As recommended in the MIQE guidelines [1], a difference of > 5 was aspired.

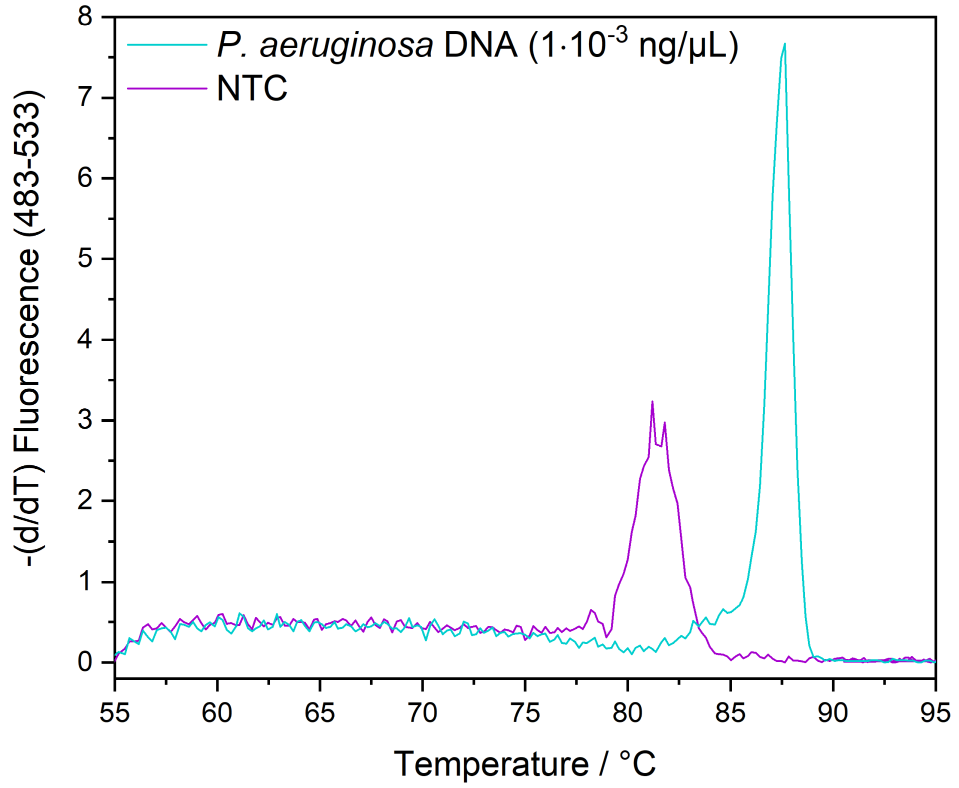

Fig. S2 Melting curves showing the formation of primer dimer in the no target control

With these considerations, a calibration range from 1 ng µL^{-1} to 0.005 ng/µL resulted. A calibration linear, recorded using six calibration concentrations in triplicates, is shown in Figure S3. For all calibrations, it was taken care that the qPCR efficiency was between 90% and 110%. For the shown calibration linear with a y-intercept of 19.4 and a slope of – 3.28, the efficiency is determined as 101.8%. The R^2 is 0.998.

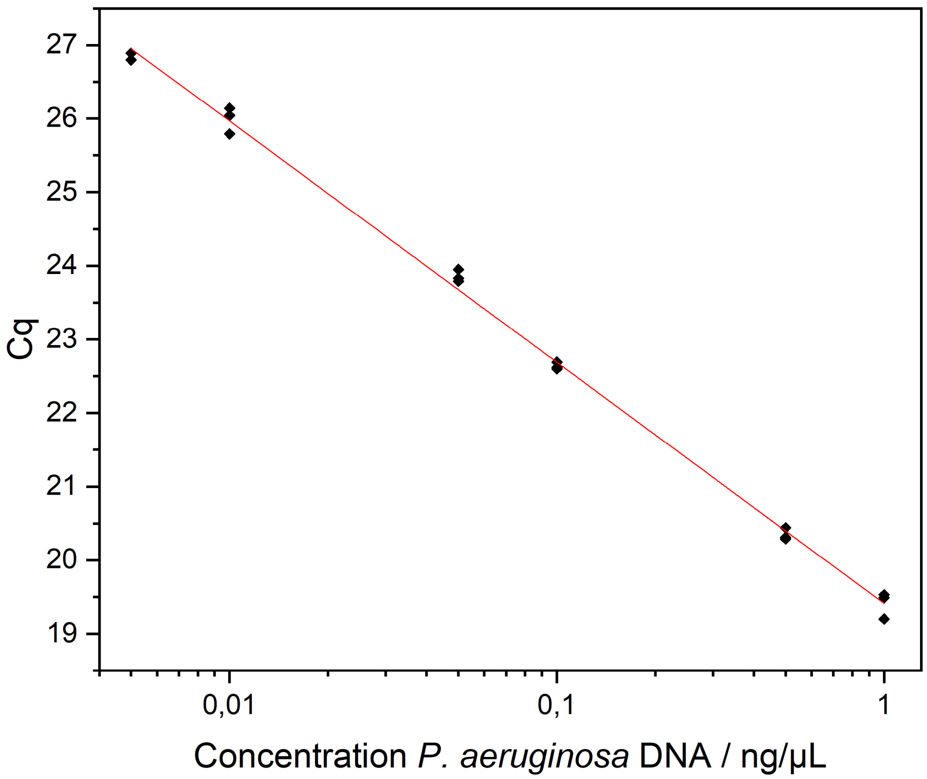

Fig. S3 Calibration linear for P. aeruginosa concentrations from 1 ng/µL to 0.005 ng/µL (n = 3)

Bacteria culture concentration calculation by OD

The concentration of *P. aeruginosa* cultures used for the spiking of tap water samples was done by optical density (OD) measurements at a wavelength of 600 nm. To obtain a calibration linear for the correlation of OD and bacteria concentration, the optical density of culture samples diluted with culture medium was measured in triplicates. Additionally, dilutions of the same culture sample were plated on agar plates in triplicates, incubated over night and counted on the following day. From the found colonies, the number of colony forming units (CFU) per mL of initial culture suspension was calculated and correlated to the previously recorded OD values of the dilutions. The resulting calibration linear is shown in Figure S4. From this calibration, the formula

$\text{CFU mL}^{-1} = (\text{OD}_{600} – 0.0033)/(1.1244 \cdot 10^8)$,

also presented in the experimental part, was obtained.

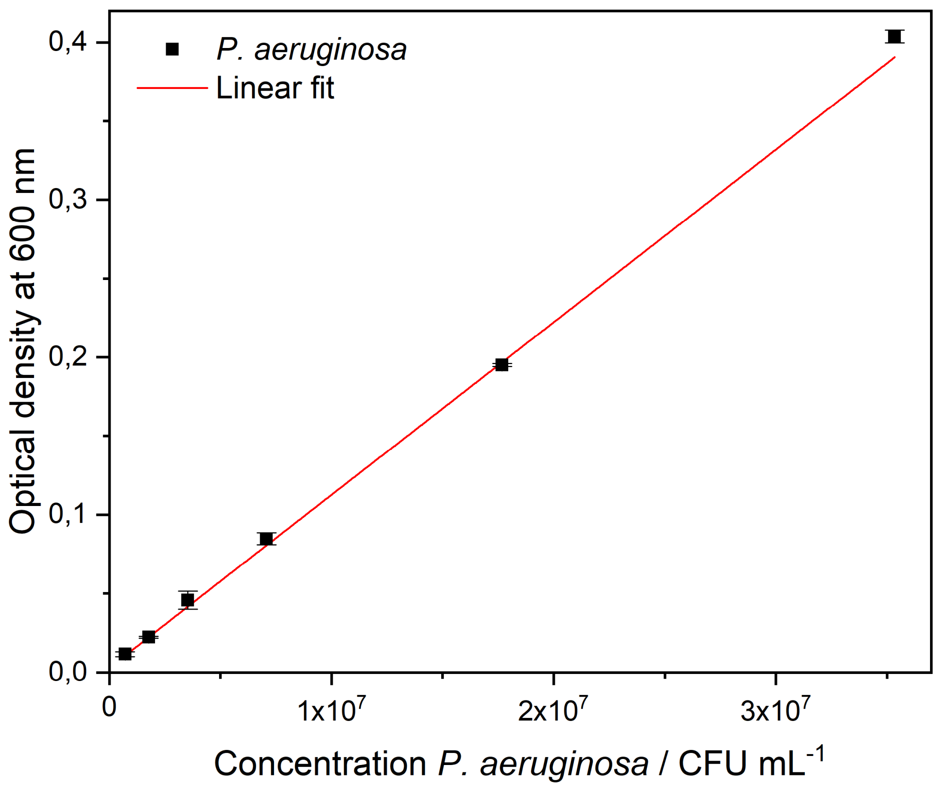

Fig. S4 Optical density calibration showing the relation between cell number (determined by culture) and optical density of culture samples (n = 3)

For the spiking of samples, the OD of the used culture was determined, and the theoretical concentration determined and used for the calculation of the necessary volume for spiking of the desired concentration. The CFU mL^{-1} of the used culture was additionally checked by plating, but the additional concentration assessment using OD was necessary to avoid the long incubation time necessary for cultivation of *P. aeruginosa* on agar plates.

Calculation of recovery values

Recovery values were calculated as ratio between the cell number found in the respective eluate samples and the total cell number spiked into the corresponding tap water sample used for filtration. To determine the cell number in eluates, qPCR was used. For the calculation of CFU from the obtained DNA concentration, a formula was developed by extracting DNA from 1 mL of culture sample and determining the DNA content in ng μL^{-1} by qPCR and plating the original sample on agar plates to assess the correct cell number in the culture. By this method, it was found that 1 ng μL^{-1} in qPCR corresponds to a number of $5.75 \cdot 10^6$ *P. aeruginosa* cells per mL of solution before DNA extraction. The cell number in the spiked samples was obtained using the aforementioned OD calibration. Recovery values are given in %.

Optimization efforts for MAF-DEAE

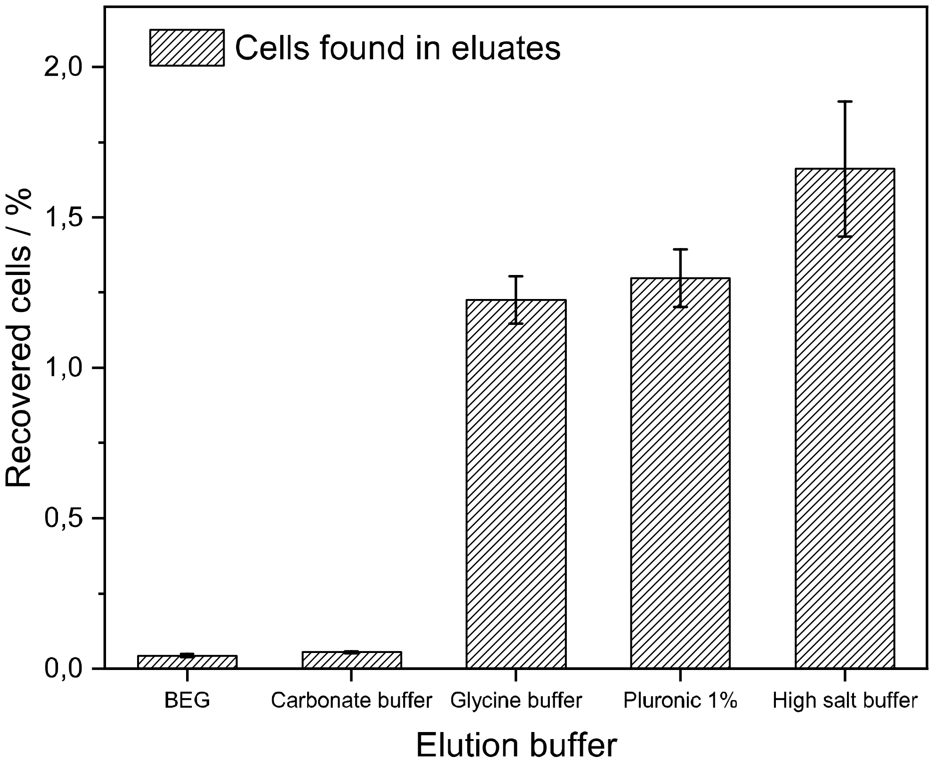

Fig. S5 Comparison of filtration of MAF-DEAE with different elution buffers with a sample volume of 1 L (n = 3)

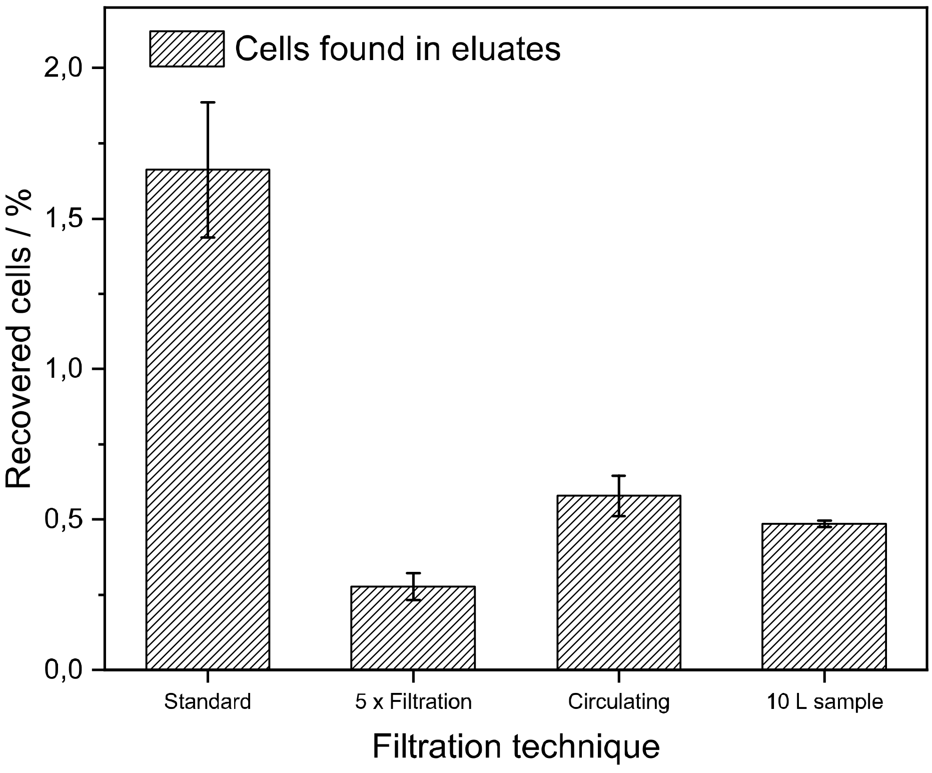

Fig. S6 Comparison of different filtration techniques for MAF-DEAE. Standard corresponds to 1 L sample, one-time filtration, 5 x filtration corresponds to filtrating a 1 L sample

repeatedly, circulating corresponds to circulating a 1 L sample for 1 h, and 10 L sample corresponds to one-time filtration of a 10 L sample and subsequent elution with high salt buffer. Sample pH = 7 (n = 3)

Different optimization efforts for MAF-DEAE were carried out. The different elution buffers BEG, carbonate buffer, glycine buffer, Pluronic® F68 solution, and high salt buffer gave recovery values of 0.04 ± 0.01%, 0.054 ± 0.003%, 1.23 ± 0.08%, 1.30 ± 0.10%, and 1.66 ± 0.23%, respectively. Additional comparison of different filtration techniques starting from the conditions that gave the best results in the elution buffer optimization (Figure S5), respectively filtration of 1 L sample and elution with high salt buffer (referred to as “standard” in Figure S6) resulted in recoveries of 1.66 ± 0.23% for the standard procedure, 0.28 ± 0.04% for the repeated filtration, 0.58 ± 0.07% for the circulating filtration, and 0.49 ± 0.01% for the 10 L sample. As all these efforts did not increase the recovery enough to be comparable to MAF-OH results, further investigation of MAF-DEAE was not carried out and resources were dedicated to increasing the MAF-OH recovery.

[1] Bustin SA, Benes V, Garson JA, et al. The MIQE guidelines: minimum information for publication of quantitative real-time PCR experiments. *Clin Chem*. 2009;55(4):611-622. doi:10.1373/clinchem.2008.112797
